# Convenient and Sensitive Measurement of Lactosylceramide Synthase Activity Using Deuterated Glucosylceramide and Mass Spectrometry

**DOI:** 10.3390/ijms24065291

**Published:** 2023-03-10

**Authors:** Michele Dei Cas, Linda Montavoci, Sara Casati, Nadia Malagolini, Fabio Dall’Olio, Marco Trinchera

**Affiliations:** 1 Department of Health Sciences, San Paolo Hospital, Università degli Studi di Milano, 20142 Milano, Italy; 2 Department of Biomedical, Surgical and Dental Sciences, Università degli Studi di Milano, 20133 Milan, Italy; 3 Department of Medical and Surgical Sciences (DIMEC), Università di Bologna, 40126 Bologna, Italy; 4 Department of Medicine and Surgery (DMC), University of Insubria, 21100 Varese, Italy

**Keywords:** glycosyltransferase, glycosphingolipid biosynthesis, enzyme activity, radiochemicals, tandem mass spectrometry

## Abstract

Lactosylceramide is necessary for the biosynthesis of almost all classes of glycosphingolipids and plays a relevant role in pathways involved in neuroinflammation. It is synthesized by the action of galactosyltransferases B4GALT5 and B4GALT6, which transfer galactose from UDP-galactose to glucosylceramide. Lactosylceramide synthase activity was classically determined in vitro by a method based on the incorporation of radiolabeled galactose followed by the chromatographic separation and quantitation of the product by liquid scintillation counting. Here, we used deuterated glucosylceramide as the acceptor substrate and quantitated the deuterated lactosylceramide product by liquid chromatography coupled with tandem mass spectrometry (LC-MS/MS). We compared this method with the classical radiochemical method and found that the reactions have similar requirements and provide comparable results in the presence of high synthase activity. Conversely, when the biological source lacked lactosylceramide synthase activity, as in the case of a crude homogenate of human dermal fibroblasts, the radiochemical method failed, while the other provided a reliable measurement. In addition to being very accurate and sensitive, the proposed use of deuterated glucosylceramide and LC-MS/MS for the detection of lactosylceramide synthase in vitro has the relevant advantage of avoiding the costs and discomforts of managing radiochemicals.

## 1. Introduction

**Figure 1:**
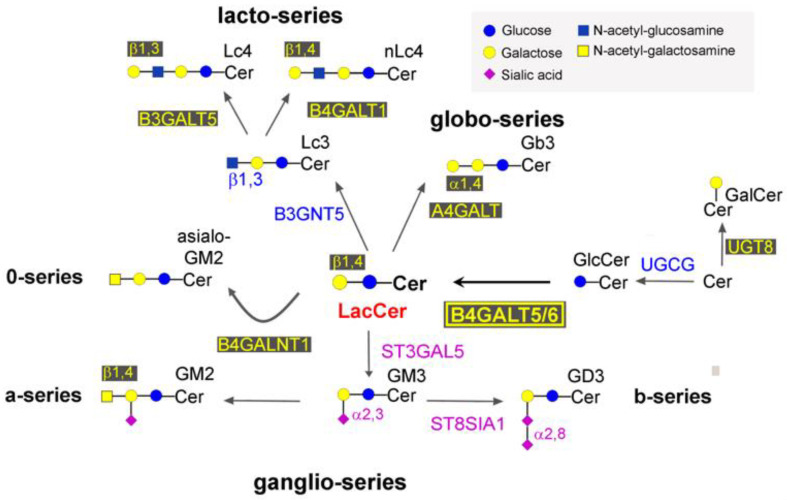
Biosynthesis of the major classes of glycosphingolipids. Monosaccharides are depicted according to the current representation. Anomers, linkage positions, and enzymes involved in the reactions are indicated. Glycosyltransferases are named according to the HUGO recommendations.

**Figure 2:**
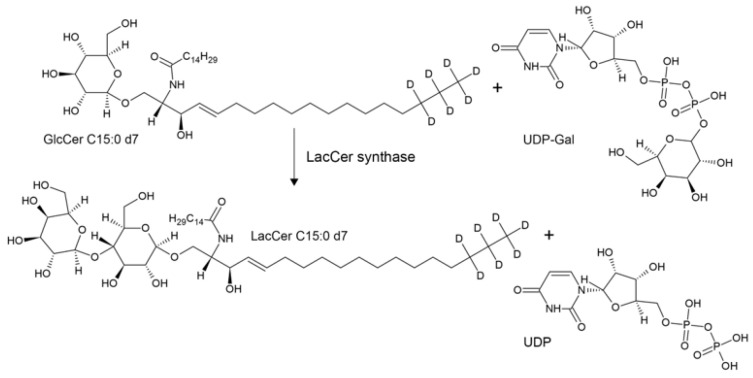
LacCer synthase catalyzed reaction. Acceptor substrate GlcCer is depicted as the deuterated molecular species used in the novel assay.

The various classes of mammalian glycosphingolipids present on cell membranes are all derived from lactosylceramide (LacCer) (Figure 1). The biosynthesis of such a key molecule depends on an enzymatic activity known as LacCer synthase (EC 2.4.1.274), which is able to transfer galactose in a β1,4 linkage from UDP-galactose to glucosylceramide (GlcCer) (Figure 2). At present, two members of the large family of B4GALTs (β1,4 galactosyltransferases) are able to act as LacCer synthases: B4GALT5 and B4GALT6 [1,2]. The two human enzymes share 70% identity at the aminoacid sequence level. The role of these two enzymes has been elucidated in mice via the phenotypic study of knock-out (KO) animals for *b4galt5*, *b4galt6*, or both. *B4galt6* KO mice were healthy [3], whereas *b4galt5* KO mice died at an early embryonic age [4], and conditional KO mice that only lacked *b4galt5* in the brain were also healthy [5]. Double KO mice, conditional *b4galt5* and *null b4galt6*, were born normally but were severely compromised in the central nervous system and died by four weeks of age [5]. Altogether, these data suggested that B4GALT5 is the major LacCer synthase [3,6] that can be rescued by B4GALT6, at least in the mouse brain. In addition to being a key intermediary in complex glycosphingolipid biosynthesis, LacCer has been reported to play a specific role in several signalling pathways, such as those orchestrated by platelet-derived growth factor (PDGF), vascular endothelial growth factor (VEGF), tumor necrosis factor-α (TNF-α), and oxidized low-density lipoprotein (LDL), thus affecting inflammation and atherosclerosis (reviewed in [7]).

Moreover, LacCer specifically synthesized by B4GALT6 was found to be responsible for mediating neuroinflammation via interplay with cytosolic phospholipase A2 in astrocytes [8,9]. B4GALT5 was found to be modulated in several cancers, including colorectal [10], breast [11], and hepatocellular carcinoma [12], as well as in acute myeloid leukemia [13]. LacCer was also reported to be involved in diabetes [14], inflammatory bowel disease [15], adipocyte differentiation [6,16], and immunological functions of neutrophils [17]. Despite LacCer synthase’s pleiotropic relevance in both physiological and pathological conditions, a convenient enzymatic assay in vitro is still lacking. The current procedure is based on the use of radioactive UDP-Gal as a donor substrate, followed by the isolation of the radioactive LacCer product by various chromatographic techniques and quantitation by liquid scintillation counting. The only alternative proposed so far relies on the fluorescent acceptor substrate NBD-glucosylceramide and the detection of the NBD-LacCer product by HPLC [18]. Such a procedure was found to be very sensitive, but the required instrumentation is not routinely available, and the fluorescent substrate is quite different from its natural counterpart. Both restrictions have limited the application of such a procedure. In the present article, we reported an approach based on the use of deuterated GlcCer as an acceptor and the detection of the deuterated LacCer product in the reaction by liquid chromatography coupled with tandem mass spectrometry (LC-MS/MS). Our aim was to set a very sensitive method, avoiding the costs and troubles of radioactive UDP-Gal manipulation and supply.

## 2. Results

### 2.1. Detection of LacCer Synthase Activity by Radiolabeled UDP-Gal and Chromatography

**Figure 3:**
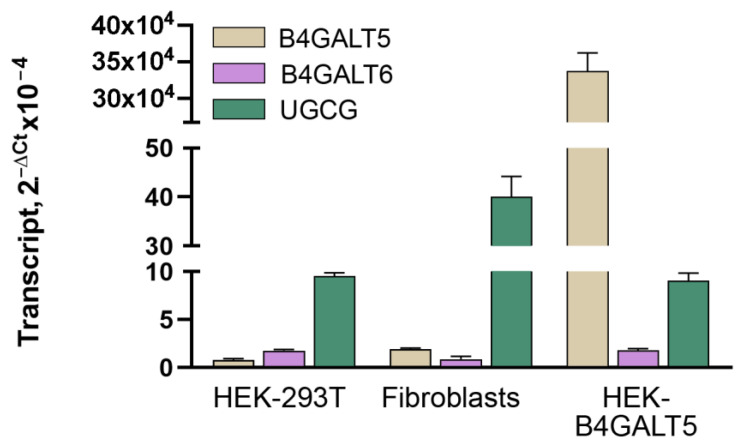
Expression of transcripts coding LacCer synthase in the cells used in this study. Quantitation of RNA transcripts was performed by real-time RT-qPCR. ΔCt values were calculated using GAPDH as the reference housekeeping gene.

**Figure 4:**
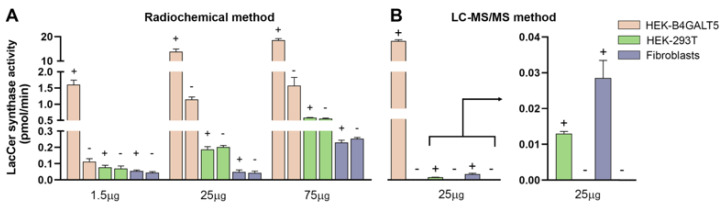
Detection of LacCer synthase activity by classical radioisotope method. Various amounts of homogenate protein prepared from 3 different sources were incubated in reaction mixtures containing tritiated UDP-Gal together with (+) or without (-) the acceptor substrate GlcCer. Quantitation was performed by using descending paper chromatography of the mixture followed by liquid scintillation counting (Panel (**A**)). True LacCer synthase activity is assumed to be the one detected in the presence (+) of GlcCer, while the values obtained in its absence (-) represent the background. For comparison, the results obtained with the same enzyme sources by the novel method were presented at a single protein concentration (Panel (**B**)).

Previous literature data have shown that LacCer synthase activity is due to the expression of B4GALT5, as well as the expression of B4GALT6 but to a lesser extent since the former is considered more efficient and relevant than the latter [5,6]. We determined the expression levels of their transcripts in the model cell line HEK-293T and in human dermal fibroblasts. We found that both transcripts are expressed at low levels in these two cell types when compared with that of the UGCG transcript (Figure 3), the one encoding GlcCer synthase, the enzyme forming the immediate biosynthetic precursor of LacCer. We thus measured LacCer synthase activity in three alternative sources: HEK-293T cells, HEK-293T cells transiently transfected with pcDNA3-B4GALT5 (named HEK-B4GALT5), and human dermal fibroblasts. Notably, upon transfection, the B4GALT5 transcript increases 10^5^-fold in HEK-293T cells (Figure 3). Using tritiated UDP-Gal as the donor substrate and unlabeled GlcCer as the acceptor substrate, strong synthase activity was detected only in HEK-B4GALT5 cells. In fact, reactions lacking the GlcCer substrate incorporated 10–20-fold less tritiated Gal than those with the complete reaction mixture (Figure 4). Conversely, in the case of both untransfected (or mock-transfected) HEK-293T and dermal fibroblasts, the incorporation of tritiated Gal was not significantly different with or without acceptor GlcCer at any tested amounts of homogenate proteins (Figure 4). As expected, LacCer synthase activity in HEK-B4GALT5 required Mn^2+^ and was slightly stimulated by CDP-Choline (not shown). 

Since LacCer synthase is a Golgi apparatus resident activity, we tried to make it measurable in fibroblasts by preparing an enriched Golgi fraction for use as the enzyme source. Starting from large amounts of cells, we prepared a subcellular fraction enriched over 20-fold in sialyltransferase and galactosyltransferase activities measured with asialofetuin or ovalbumin as acceptor substrates, respectively. Using such a fraction as the enzyme source, we measured a LacCer synthase activity of 7.5 pmol/mg/min (Table 1), confirming that the activity was actually present in fibroblasts. Due to low expression levels, the assay’s procedure was unable to detect LacCer synthase in the crude homogenate. 

**Figure 5:**
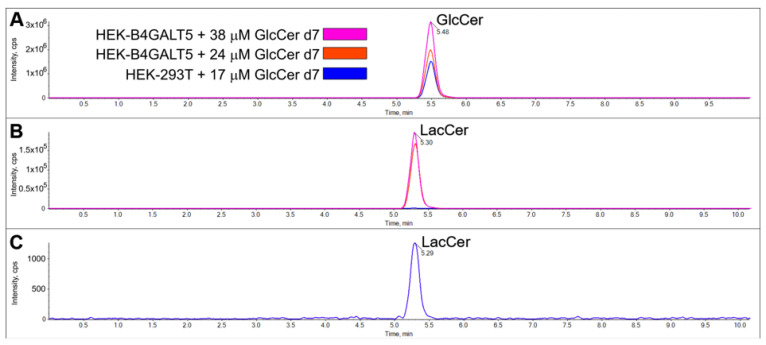
Detection of LacCer synthase activity by the novel method using deuterated glucosylceramide (GlcCerd7). The enzymatic conversion to lactosylceramide (LacCerd7) was monitored by LC-MS/MS. Comparison of GlcCerd7 (**A**) and LacCer (**B**) profiles in HEK-B4GALT5 and HEK-293T cells. Since it appears as a baseline with the scale used in this last case, it is presented alone in panel (**C**) with a different scale. To obtain these chromatograms, various amounts of GlcCerd7 with 0.15 mg/mL of cell homogenate (expressed as proteins) were incubated for 60 min. Note the different scales used in each panel; cps, counts per second.

The attempt to measure LacCer synthase activity in vitro using GlcCerd7 as the acceptor substrate and unlabeled UDP-Gal as the donor substrate was performed under the same reaction conditions as in the radioisotopic assay, with the HEK-B4GALT5 homogenate as the enzyme source. As a negative control, we prepared reaction mixtures lacking GlcCerd7 or containing homogenate from untransfected HEK-293T cells. By LC-MS/MS, a peak corresponding to LacCerd7 was detected in the reaction mixture containing HEK-B4GALT5 homogenate that appeared negligible with mock-transfected cells, being more than 200-fold lower (Figure 5, note the different scales used in each panel). 

**Figure 6:**
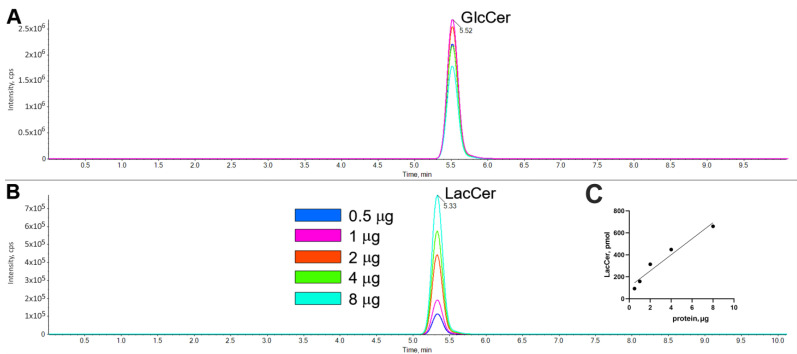
Effect of protein amounts on the enzymatic conversion of GlcCerd7 to LacCerd7 monitored by LC-MS/MS. Comparison of GlcCerd7 (**A**) and LacCerd7 (**B**) profiles using HEK-B4GALT5 as the enzyme source. To obtain these chromatograms, 40 µM of GlcCerd7 with cell homogenates ranging from 0.5 to 8 µg (expressed as proteins) was incubated for 60 min in a reaction volume of 20 μL. The amounts of LacCer formed at each protein concentration is plotted in subpanel (**C**).

**Figure 7:**
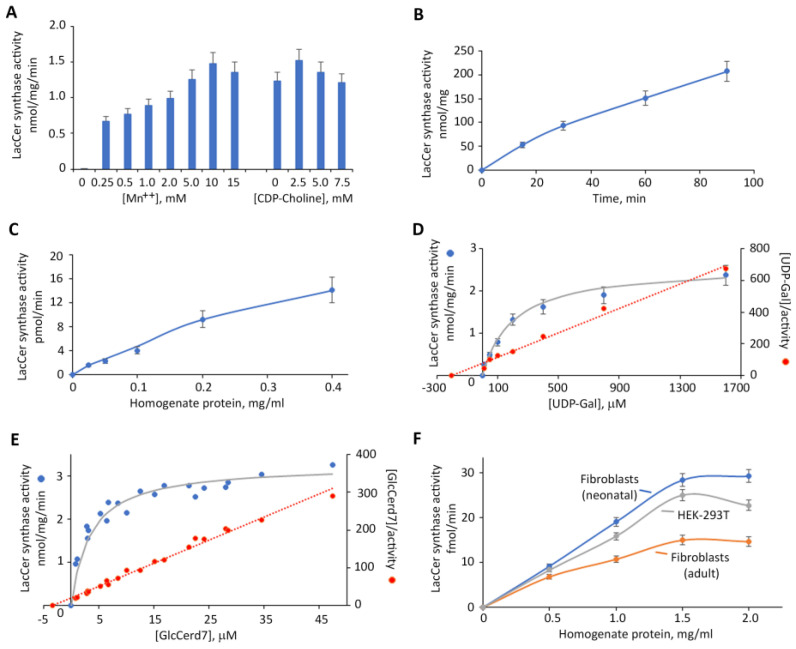
Characterization of LacCer synthase activity detected with GlcCerd7 and LC-MS/MS. Dependence of the activity expressed in HEK-B4GALT5 on the concentration of Mn^++^ or CDP-Choline (Panel (**A**)). Activity measured in HEK-B4GALT5 after different times of incubation (Panel (**B**)) or using different amounts of homogenate protein (Panel (**C**)). Dependence of the activity expressed in HEK-B4GALT5 on the concentration of the donor substrate UDP-Gal at fixed saturating concentration of GlcCerd7 (10 μM). The Hanes–Woolf plot of activity values, presented in red (secondary axis), was used for calculating kinetic constants (Panel (**D**)). Dependence of the activity expressed in HEK-B4GALT5 on the concentration of the acceptor substrate GlcCerd7 at a fixed saturating concentration of UDP-Glc (0.6 mM). The Hanes–Woolf plot is shown in Panel D (Panel (**E**)). The reactions reported in Panels D and E were obtained by incubating 0.5 μg of HEK-B4GALT5 homogenate protein for 25 min, which assured initial rates. Detection of the activity in crude homogenates of cells expressing low levels of B4GALT5/6 transcripts using different amounts of homogenate protein (Panel (**F**)).

The enzymatic conversion of GlcCerd7 to LacCerd7 was linear with the amount of HEK-B4GALT5 homogenate (Figure 6 and Figure 7 panel C). In the absence of GlcCerd7, no peak at all at the size of LacCerd7 was detectable at any homogenate protein concentration.

### 2.2. Characterization of LacCer Synthase Activity Detected by GlcCerd7 and LC-MS/MS

**Figure 8:**
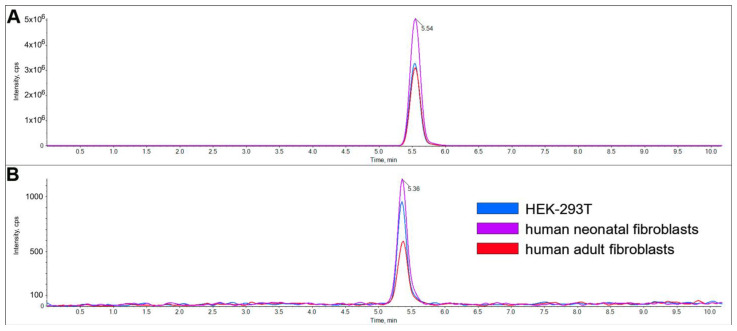
Detection of LacCer synthase by the novel method in cells expressing low levels of activity. Comparison of GlcCerd7 (**A**) and LacCerd7 (**B**) profile in HEK-293T and fibroblasts. To obtain these chromatograms, 40 µM of GlcCerd7 with 1 mg/mL of homogenate protein was incubated for 60 min.

LacCer synthase activity in HEK-B4GALT5 cells assayed in vitro using GlcCer d7 and LC-MS/MS was found to be completely dependent on Mn^2+^, with an optimal concentration of 10 mM, and slightly stimulated by CDP-Choline, with an optimal concentration of 2.5 mM (Figure 7 panel A). The reaction was linear up to 60 min of incubation (Figure 7 panel B) and had homogenate protein concentrations up to 0.2 mg/mL (Figure 7 panel C). The activity was saturated at a UDP-Gal concentration over 0.8 mM (Figure 7 panel D), showing an apparent K_m_ value 205 μM for the donor, and by a GlcCerd7 concentration over 10 μM (Figure 7 panel E), with an apparent K_m_ of 3.37 μM for the acceptor. The corresponding Km values calculated via the non-linear regression of the Michaelis–Menten equation were 214.4 (95% confidence interval, 149.1–308.0) for the donor and 2.47 (95% confidence interval, 1.88 to 3.19) for the acceptor. Under such optimized conditions, LacCer synthase activity was also determined using crude homogenates prepared from non-transfected HEK-293T or human fibroblasts as the enzyme source. LacCer synthase activity was found to be linear up to 1.5 mg/mL homogenate protein in both fibroblasts and HEK-293T cells (Figure 7 panel F). The calculated specific activity ranged from 0.5 to 1.0 pmol/mg/min, being highest in neonatal fibroblasts undergoing less than 5 passages, lowest in adult fibroblasts with more than 15 passages, and intermediate in HEK-293T cells (Figure 8). A direct comparison of this new method with the classical radiochemical method was performed using HEK-B4GALT5 and HEK-293T and with dermal fibroblasts as the enzyme source at the same fixed protein concentration (Figure 4). LacCer synthase activity was detectable by both methods and with comparable values only in HEK-B4GALT5 cells that express very high activity. Conversely, in HEK-293T cells and dermal fibroblasts, the activity was much lower and detected by the new method only. In fact, at such low activity levels, the background of the radiochemical method overcomes the true activity value. The calculated precision, limit of detection, and limit of quantification of both methods are presented in Table 2.

## 3. Discussion

We have set a novel assay procedure for determining LacCer synthase activity in biological samples based on the conversion of deuterated GlcCer (GlcCerd7) in LacCerd7 that is then quantitated by LC-MS/MS. Compared with the classical radiochemical assay, the new procedure requires the same reaction conditions, and it provides comparable results but offers several advantages. First, it avoids the costs and all the problems associated with the manipulation of radioisotopes. In addition, GlcCerd7 is an isotope of GlcCer without the relevant modifications present in the fluorescent substrate previously proposed as alternative to the radiochemical method [18]. Moreover, the procedure appeared even more sensitive than the classical one, allowing us to measure LacCer synthase activity in crude homogenates of human fibroblasts or model cell lines in which the radioisotopic method failed. In fact, it was necessary to prepare an enriched Golgi fraction to detect the activity with such a method. Our data indicated that the radiochemical assay performed in biological sources that poorly express LacCer synthase activity results in high background incorporation. This is probably due to the presence of several endogenous unspecific substrates and other galactosyltransferases. Conversely, the same sources lack lipid compounds with a molecular mass identical to that of LacCerd7, making the background close to zero. Notably, in a 20-fold enriched Golgi fraction from fibroblasts, the specific activity of LacCer synthase, determined with the radiochemical method (7.5 pmol/mg/min), corresponds to a value about 20-fold higher than that measured in crude homogenates using the novel approach (0.5 pmol/mg/min). Coupling such high specificity with the high sensitivity of LC-MS/MS, the procedure proposed here provides an unprecedented tool for functional studies to address the role of B4GALT5/6 and the cognate LacCer product in several pathological and physiological conditions relevant to current research topics [7]. Moreover, the LC-MS/MS analysis of the reaction mixture is able to simultaneously characterize and quantify both the enzymatic product LacCerd7 and the substrate acceptor GlcCerd7, making each point value more accurate and replicates more comparable. The use of deuterated substrates is emerging as a promising alternative for measuring in vitro the activity of enzymes involved in glycosphingolipid biosynthesis, as we reported very recently for UGCG [19].

## 4. Materials and Methods

### 4.1. DNA Constructs

Human B4GALT5 and B4GALT6 cDNAs were obtained by PCR using total RNA extracted from Huh-7 or Hep-3B cells [20] in the presence of Phusion High-fidelity Taq polymerase (ThermoFischer Scientific, Rome, Italy, Italian distributor), according to the manufacturer’s protocol. The following primer pairs were used: B4GALT5 (forward) 5′-CGCG*AAGCTT*GCGATCGCCATGCGCGCCCGCCGGG and (reverse) 5′-CGCG*TCTAGA*GTTTAAACTCAGTACTCGTTCACCTGAGCC; and B4GALT6 (forward) 5′-CGCG*AAGCTT*GCGATCGCCATGTCTGTGCTCAGGCGGATG and (reverse) 5′-CGCG*TCTAGA*GTTTAAACTTAATAGTCTTCGATTGGAGCTAACTC, both containing HindIII and XbaI restriction sites (italicized), respectively. Annealing was at 64 °C, and 30 cycles of amplification were run. The obtained fragments were purified by spin columns, digested with HindIII, repurified, digested with XbaI, purified again, and ligated to the pcDNA3 vector that was previously digested/purified with the same enzymes. *E. coli* Max-efficiency DH5α (ThermoFischer Scientific) was transformed by an aliquot of ligation reactions, and the obtained colonies were inoculated in liquid cultures to prepare plasmid DNA. The obtained clones were first assessed by restriction digestion and then confirmed by direct DNA sequencing.

### 4.2. Cell Culture, Transfection, and Processing

HEK-293T cells were grown in DMEM supplemented with 10% foetal bovine serum and transiently transfected with plasmid DNA in the presence of Fugene-HD (Promega, Madison, WI, USA), as previously reported [21].

Upon transfection, cells were harvested by trypsinization, pelleted, washed twice with PBS, and aliquoted. A small aliquot (<0.5 × 10^6^ cells) was used for total RNA extraction using a Relia-Prep RNA kit (Promega), and the remaining cells (2–10 × 10^6^ cells) were resuspended in a 0.1 M Tris/HCl buffer, pH 7.5, containing 0.5% TritonX-100, vortexed to homogeneity, and stored in aliquots at −80 °C. After thawing on ice, the crude homogenate was used as the enzyme source for the in vitro assay. Protein content was determined by the bicinconic acid method (BCA protein kit, ThermoFischer Scientific). Homogenates for enzyme assay were kept at a protein concentration of about 10–20 mg/mL.

Human neonatal (P10857) and adult (P10858) fibroblasts were purchased from Innoprot (Bizkaia, Spain) and cultured in DMEM supplemented with 10% foetal bovine serum and antibiotics. Cell harvesting and aliquot preparation were performed, as was the case for transfected HEK-293 T cells.

### 4.3. Reverse Transcription Quantitative Real-Time Polymerase Chain Reaction

First strand cDNA was synthesized from 0.5 to 1 μg of total RNA by a commercial kit (GoScript oligodT mix, PromegaItalia srl, Milano, Italy). Control reactions were prepared by omitting the reverse transcriptase in the reaction. cDNAs were diluted 1:4, *v*/*v*, with mQ water, and 1–2.0 μL of first strand reactions was amplified in a volume of 20 μL using Sybr Premix Ex Taq (Tli RNase H Plus, Takara, Sandiano, Italy, Italian distributor), ROX as the reference dye, and a StepOnePlus instrument (Applied Biosystem Life Technologies, Waltham, MA, USA) as reported [22]. Primer sequences for amplification were as follows: B4GALT5 (forward) 5′-GAGAACAATCGGTGCTCAGG, (reverse) 5′-GGGCCCTTCATGGAAGGG; B4GALT6 (forward) 5′-CCGGAAAACTTCACATACTCAC, (reverse) 5′-GAACTGCCACCTTCCATCTG; UGCC (forward) 5′-GTGATAGTGGAATAAGAGTAATTCC, (reverse) 5′-TGAAGTTCCAAAATATACCTGCTC. Annealing temperature was 60 °C. The amounts of amplified target cDNAs were calculated as ΔCt with respect to GAPDH [22].

### 4.4. LacCer Synthase Activity Assay

GlcCer, purified as reported [23], or GlcCerd7 (C15 Glucosyl(β) Ceramide-d7 (d18:1-D7/15:0, 330729P-1MG], Merck, Rome, Italy, Italian distributor), with 20–800 pmol dissolved in chloroform/methanol, 2:1 (*v*/*v*), and 15 μg Triton-X100 in the same solvent were placed together at the bottom of a 0.6 mL microcentrifuge tubes and allowed to dry at RT under hood and then kept at −20 °C until used. A reaction solution was prepared and added to each tube in order to obtain the following final concentrations: 0.2 M Tris/HCl pH 7.0, 0.4 mM UDP-Gal, 10 mM MnCl2, and 2.5 mM CDP-choline. In the case of the radio isotopic method, UDP-Gal was brought to a specific radioactivity of 10 mCi/mmol by adding UDP-[3H]Gal [24]. In an ice bucket, various amounts of protein (see results) were added to each tube, which already contained water, to a final volume of 20 μL. The reactions were started by placing the tubes at 37 °C and incubating them for 10–60 min. The reactions were stopped by placing the tubes on ice and then storing them at −20 °C. In the case of the radio isotopic method, the mixture was assayed by descending chromatography, and radioactivity was measured by liquid scintillation as reported [21]. 

### 4.5. Reaction Product Characterization and Quantification by LC–MS/MS

Samples for the determination of LacCer synthase activity were precipitated by the addition of pure methanol (75 μL) and centrifuged at 13,400 rpm for 10 min. The precipitates were discarded, and pure extracts (5 μL) were directly injected in LC-MS/MS. The samples were analyzed by a high-sensitivity LC–MS/MS consisting of a QTrap 5500 triple quadrupole linear ion trap mass spectrometer (Sciex, Darmstadt, Germany) equipped with an electrospray ionization (ESI) source and coupled with an Agilent 1200 Infinity pump Ultra High-Pressure Liquid Chromatography (UHPLC) system (Agilent Technologies, Palo Alto, CA, USA). Chromatographic separation was carried out on a reverse-phase Acquity UPLC BEH C8 column at 1.7 µm particle size, and100 × 2.1 mm (Waters, Franklin, MA, USA) at 30 °C using linear gradient elution with two solvents: 0.2% formic acid and 2 mM ammonium formate in water (solvent A) and 0.2% formic acid and 1 mM ammonium formate in acetonitrile (solvent B). Solvent A and B were 20% and 80% at 0.00 min, respectively. Solvent B increased to 90% from 0.00 to 3.00 min, held at 90% from 3.00 to 6.00 min, increased to 99% from 6.00 to 10.00 min, held at 99% from 10.00 to 12.00 min, and then decreased back to 80% from 12.00 to 12.10 and held at 80% until 15.00 min for re-equilibration. The flow rate was kept constant at 0.40 mL/min during the analysis. The separated analytes were detected with a triple quadrupole MS operated in multiple reaction monitoring (MRM) mode via a positive ESI using the following precursor ion and product ions transition: GlucCerd7 C15:0 (*m*/*z* 693.6 > 271.3, CE 45 eV, DP 65 eV) and LacCer d7 C15:0 (*m*/*z* 855.6 > 271.3, CE 60 eV, DP 65 eV). Data acquisition and processing were performed using Analyst^®^1.7.1 and MultiQuant^®^2.1.1 software (Sciex, Darmstadt, Germany), respectively. The declustering potential (DP) and collision energy (CE) are compound-dependent parameters that should be investigated before every mass spectrometry experiment. In particular, DP is a voltage applied—in MS ion source—that helps prevent ions from clustering together, whereas CE is the voltage produced to induce the dissociation of the molecular ion from their fragments’ ions. One of the most popular targeted types of MS experiments is multiple reaction monitoring (MRM). With the use of a triple quadrupole mass spectrometer, this method first targets the interested protonated form of the molecular ion before fragmenting it to create different daughter ions depending on the applied CE.

### 4.6. Preparation of Golgi Apparatus Fraction from Fibroblasts

About 2.5 × 10^7^ fibroblasts were detached by trypsinization, washed twice with PBS, resuspended with 0.3 mL of 10 mM Tris/HCl pH 7.5 containing 0.1 mM EDTA and 0.25 M sucrose, and homogenized by passing them several times through a 27G needle of a 1.0 mL syringe. The obtained homogenate was spun at 2000 g for 10 min at 4 °C. The supernatant was removed, and the pellet was rehomogenized and spun as above. The two supernatants were combined, 40% sucrose was made in the same buffer (final volume about 1.4 mL) and placed at the bottom of an ultracentrifuge tube and overlayered with 1 mL each of 35% and 25% sucrose solutions prepared in the same buffer, and they were covered with 0.25 M sucrose solution. The tube was spun at 54,000 rpm in a Beckmann Ti 55 rotor for 90 min at 4 °C. Materials migrating at the 35–25% sucrose interface were collected, diluted 1:8 (*v*/*v*) with PBS, and spun again at 54,000 rpm in a Beckmann Ti 55 rotor for 60 min at 4 °C to obtain the Golgi membrane fraction that was resuspended with 0.05 mL of 0.1 M Tris/HCl pH7.0 containing 0.2% Triton-X100. Sialyltransferase activity with asialofetuin and galactosyltransferase activity with ovalbumin were determined, as reported with other glycoprotein substrates [25].

### 4.7. Equations and Statistical Analysis

LacCer synthase catalyzes a reaction that uses two substrates (GlcCer and UDP-Gal) and forms two products (LacCer and UDP). Consequently, Michaelis–Menten kinetics could be used only by keeping either substrate at saturating concentrations and varying the concentrations of the other. For a graphical description and apparent kinetic constant calculations, the Hanes–Woolf equation was used: [S]/v = 1/V_maxapp_ * [S] + K_mapp_/V_maxapp_. Linear regression was obtained via Microsoft Excel. *R*^2^ values were 0.990 (UDP-Gal saturation curve) and 0.982 (GlcCerd7 saturation curve). Non-linear regression of the Michaelis–Menten equation was obtained via GraphPad Prism (Dotmatics Scientific, Boston, MA, USA). The specificity of LacCer synthase assays was assessed by using the response of a blank sample at the limit of quantitation (LOQ). The LOQ was determined as the lowest concentration with a signal-to-noise ratio of the instrumental response ≥10; the limit of detection (LOD) was determined as the lowest concentration with a signal-to-noise of ≥3. Precision of the methods was determined by assaying six biological replicates and calculating the coefficient of variation (CV%), which should be appropriate if it is lower than 15%. For transcripts quantitation, qPCR was performed in duplicate twice, starting from cDNA prepared from two independent transfections of HEK-293T cells or fibroblast cultures. For LacCer synthase activity determination, assays were performed in duplicate twice, starting from two individual cell homogenates consisting of distinct cell plates of growing cells or independent transfections of HEK-293T cells.

## Figures and Tables

**Table 1:** LacCer synthase activity determined by the classical radioisotope method in a Golgi fraction prepared from human dermal fibroblasts.

	Total Protein		Ovalbumin Galactosyltransferase			Asialofetuin Sialyltransferase			LacCer Synthase
	μg	(%)	Specific Activity	(RSA)	Recovery %	Specific Activity	(RSA)	Recovery %	Specific Activity
Total homogenate	5670	100	16.7	1	100	10.5	1	100	ND
Golgi fraction	93.6	1.65	337	20.2	33.3	300	28.5	47.1	7.5

Specific activity is expressed as pmol/mg/min. Values are the mean for triplicate assays performed on a single large-scale subcellular fractionation. Standard deviations were always below 15% of the mean value. RSA, relative specific activity; ND, not detectable.

**Table 2:** Comparison between method performances: liquid chromatography coupled to mass spectrometry (LC-MS/MS) vs. radiochemical method. The comparison was based on analytical parameters such as the limit of detection (LOD), limit of quantification (LOQ), and precision (calculated as coefficient of variation (CV%).

	LOD	LOQ	CV% LacCer	CV% GlcCer
	pmol/mg × min	pmol/mg × min	n = 6	n = 6
LC-MS/MS	0.05	0.2	3.34	5.07
Radiochemical method	100	330	10.7	-

## Abbreviations

B4GALT	β1,4 galactosyltransferases (according to HUGO nomenclature)
CE	collision energy
DP	declustering potential
GlcCer	glucosylceramide
GlcCerd7	deuterated glucosylceramide
LacCer	lactosylceramide
LacCerd7	deuterated lactosylceramide
LC-MS/MS	liquid chromatography coupled with tandem mass spectrometry
KO	knock-out
NBD—ceramide	6-((*N*-(7-Nitrobenz-2-Oxa-1,3-Diazol-4-yl)amino)hexanoyl)Sphingosine
RSA	relative specific activity
UGCG	UDP-glucose: ceramide glucosyltransferase
